# Enhancing maternal health in Zambia: a comprehensive approach to addressing postpartum hemorrhage

**DOI:** 10.3389/fgwh.2024.1362894

**Published:** 2024-08-06

**Authors:** Mulaya Mubambe, Job Mwanza, Enos Moyo, Tafadzwa Dzinamarira

**Affiliations:** ^1^ Department of Obstetrics and Gynaecology, Levy Mwanawasa University Teaching Hospital, Lusaka, Zambia; ^2^ ICAP in Zambia, Lusaka, Zambia; ^3^ Department of Public Health Medicine, University of KwaZulu-Natal, Durban, South Africa; ^4^ School of Health Systems and Public Health, University of Pretoria, Pretoria, South Africa

**Keywords:** postpartum hemorrhage, maternal health, interventions, challenges, opportunities

## 1 Introduction

Maternal mortality remains a pressing concern globally, with postpartum hemorrhage (PPH) being a significant contributor. PPH remains a significant public health challenge in Zambia, contributing to 34% of maternal deaths, as per a recent study (1). Zambia is a country in Southern Africa with a population of about 20 million. About 60% of the Zambian population lives in rural areas. 60% of Zambians were considered impoverished in 2022, making it one of the nations with the greatest rates of poverty (2). Due to logistical and physical obstacles like high transportation costs and extensive travel distances, access to healthcare services is restricted in rural areas of the country (3). This high prevalence of PPH can be attributed to several factors, including high parity, limited antenatal care (ANC), skilled birth attendant (SBA) shortage, and socio-economic constraints to mention a few (4).

Women in Zambia often experience multiple pregnancies, with uterine fatigue increasing the risk of PPH (5). Secondly, inadequate access to prenatal screening and interventions may increase vulnerability to complications during childbirth (6). This coupled with a lack of sufficient SBAs, particularly in rural areas, compromises the quality of delivery care and timely identification of PPH risks. Regarding socio-economic challenges, poverty can restrict access to essential maternal healthcare services (7), including blood transfusions and surgical procedures, crucial for managing severe PPH.

This opinion manuscript explores the current landscape of PPH management in Zambia, highlighting the Zambian Ministry of Health’s initiatives and partnerships and proffers a perspective on opportunities for strengthening efforts to address PPH in the country.

## 2 Current PPH priorities in Zambia

### 2.1 Blood transfusion services related challenges

The shortage of blood and blood products, coupled with delays in access, stands out as a major obstacle in managing PPH cases in Zambia (1). Zambia requires 180 000 blood units annually to cover its demand for blood and blood products. Nonetheless, between 2017 and 2021, only 110 000 blood units were collected each year (8). According to estimations from the World Health Organization (WHO), 1% of the population must donate blood in order to meet the nation's basic blood needs. Zambia, however, has not been able to satisfy the WHO's predicted requirement of one percent of the population (8). The 10 blood transfusion centers, strategically located in provincial hospitals, face difficulties meeting the demand from remote health facilities. To bridge this gap, the Ministry of Health is implementing a multifaceted strategy in collaboration with partners.

#### 2.1.1 Structural improvements

The Provincial blood transfusion centers play a pivotal role in collecting, processing, testing, and distributing blood and blood products. The Ministry aims to bolster these centers by investing in capital infrastructure, including equipment procurement and staff training programs (9). The Ministry of Health is committed to ensuring self-sufficiency in blood and blood products by making sure that the country reaches the blood donation rate of one percent recommended by the WHO (10). The Ministry of Health's investment in capital infrastructure for blood transfusion centers is projected to yield several positive outcomes in the fight against PPH. These include increased blood collection and processing capacity, enhanced blood safety, improved efficiency and turnaround time, and setting the ground work for potential automation.

#### 2.1.2 Hub system implementation

The Ministry of Health established hubs in two districts per province, strategically positioning already processed blood closer to communities (8). This decentralized storage model aims to minimize delays in delivering blood to where it is needed, thereby addressing accessibility challenges.

#### 2.1.3 Communication strategy

Creating awareness within the community is paramount to ensure a steady supply of blood. The Ministry of Health routinely conducts comprehensive awareness campaigns, educating the public on the demand for blood and the critical role they play in donating (8).

#### 2.1.4 Clinical interface improvements

Recognizing the need for skilled clinicians in conducting blood transfusions, the Ministry of Health is initiating targeted training programs (8). These programs aim to enhance clinical skills, ensuring safe and effective blood transfusions, consequently reducing the waiting time for patients in need.

#### 2.1.5 Blood transfusion camps

Despite facing resource constraints, the Ministry of Health organizes blood transfusion camps to mobilize communities, including schools, for voluntary blood donations. While resource limitations pose challenges, these camps have been useful in propelling the country to align with the WHO requirements and contribute to building a sustainable supply of blood.

### 2.2 Surgical skills for early intervention

In addressing the challenge of PPH management and general maternal health service provision, the Ministry of Health in Zambia acknowledges the indispensable role of surgical interventions. The scarcity and uneven distribution of obstetric consultants across the country stand out as a critical issue, resulting in delayed interventions and tragic outcomes (11, 12). Some patients with PPH requiring definitive management such as hysterectomy must be referred to a Provincial hospital resulting in delayed intervention and death (5). To bridge this gap, the Ministry of Health is implementing a multifaceted strategy that includes:

#### 2.2.1 Abridged specialty training program

To confront the scarcity of obstetric specialists, particularly in remote areas, the Zambian government has launched an innovative abridged specialty training program (8). This initiative is designed not only to augment the overall number of obstetric consultants but also to strategically place them in district hospitals. The goal is to facilitate swift and effective surgical interventions for managing PPH at the local level, addressing the unique challenges posed by low-resource settings.

#### 2.2.2 Task shifting and community engagement

Recognizing the resource constraints in many district hospitals, the Ministry is exploring task-shifting strategies (8). This involves training and empowering healthcare workers, beyond traditional obstetric consultants, to perform specific surgical interventions for PPH. A study conducted in Malawi to determine postoperative outcomes of patients after caesarean section and other emergency obstetric surgeries performed by clinical officers and medical officers revealed that there were similar postoperative outcomes (13). Additionally, community engagement initiatives are underway to create a network of support and awareness, emphasizing the importance of early intervention in managing PPH.

### 2.3 Antenatal care (ANC) service provision quality

Quality antenatal care remains a cornerstone in the prevention and early identification of high-risk pregnancies for PPH. However, in low-resource settings, additional challenges arise, necessitating targeted interventions to enhance ANC quality. There is a need to improve the skills of staff to recognize high-risk pregnancies for PPH with timely referral for management at an appropriate level of care in Zambia (14). A common challenge faced is cases when a previous cesarian delivery patient is being managed at a health center in a rural area distant from a hospital and only referred for delivery when in labor-these mostly tend to have a ruptured uterus and a high risk of dying if the hospital does not have a skilled staff to manage a ruptured uterus (4). To bridge the gap in quality of ANC, the Ministry of Health is implementing a multifaceted strategy that includes:

#### 2.3.1 Provision of ANC cards to pregnant women

The Ministry of Health has undertaken an approach to provide each pregnant woman with an ANC card. ANC cards are updated at each contact, which allows for an accurate record of a woman's pregnancy (8, 15). The Ministry of Health continues to upgrade the design or content of the cards to capture more comprehensive information and train healthcare providers on the optimal use of ANC cards for risk identification and communication with pregnant women. This is expected to improve the continuity and quality of ANC care, as well as the pregnant women's experience of pregnancy. Furthermore, ANC cards may improve health awareness among pregnant women and communication between pregnant women and healthcare providers.

#### 2.3.2 Strengthening referral mechanisms

To improve the referral of pregnant women who need the care of specialists, the Ministry of Health devised several mechanisms. One of the mechanisms is the development of referral protocols and guidelines for healthcare providers to use. While it might be challenging to quantify the precise impact of referral protocols on timeliness of care with available data, their implementation is a crucial step towards ensuring that high-risk pregnancies are identified and referred promptly. Studies conducted in India showed that establishing clear referral protocols led to a significant increase in the number of women receiving timely specialist care for high-risk pregnancies (16). The Ministry is also trying to ensure that there are reliable and readily available well-equipped ambulances to transport referred pregnant women between healthcare facilities (8, 15).

## 3 Opportunities for improvement

Although several strategies are being implemented to reduce the outcomes of PPH, there are some areas that need improvement. Blood transfusion-related challenges need to be addressed while the quality of ANC service should be improved.

### 3.1 Addressing blood transfusion-related challenges

The concerted efforts by the Ministry of Health to enhance blood transfusion services are promising steps toward reducing maternal mortality due to PPH. The strategic interventions outlined demonstrate a commitment to overcoming logistical challenges and ensuring a timely and sufficient supply of blood and blood products. However, some opportunities for improvement exist.

To increase blood donation, blood bank organizations should dispel myths and religious beliefs about blood donation. This can be achieved by strengthening community involvement in developing communication messages on the importance of blood transfusion. Where donation centers are located far from potential donors, the organizations should conduct outreach programs to target potential donors. Furthermore, organizations should create trusting relationships with donors so that they can keep donating blood in the future (17).

Furthermore, recent advancements in drone technology offer exciting possibilities for revolutionizing blood product delivery, particularly in remote areas with limited access to healthcare facilities. Studies have shown that drones can deliver blood products efficiently and rapidly, significantly reducing transportation times compared to traditional methods (18). This technology holds immense potential to address logistical challenges and ensure timely access to blood transfusions for women experiencing PPH, especially in geographically dispersed regions of Zambia. Integrating drone delivery systems into the Zambian Ministry of Health's existing blood transfusion strategies could significantly improve maternal health outcomes and contribute to a reduction in PPH-related deaths.

### 3.2 Ensuring ANC service quality

The Ministry of Health has undoubtedly led promising efforts to improve ANC service quality in the country. We suggest some opportunities for strengthening already existing efforts.

#### 3.2.1 Community-based ANC initiatives

Recognizing the limitations of rural health centers, the Ministry of Health can strengthen community-based ANC initiatives. Community-based ANC initiatives such as trained community health workers hold immense potential to improve access and quality of care. These workers can be equipped to conduct basic pregnancy screenings and identify potential risk factors through monitoring blood pressure, weight gain, and other key indicators. Furthermore, they can be trained to deliver essential ANC services in remote areas, including providing prenatal vitamins, administering vaccinations, and offering education on healthy pregnancy practices. Finally, community health workers can play a vital role in referral and care coordination by effectively linking high-risk pregnant women with appropriate healthcare facilities at district hospitals or higher levels of care, facilitating communication and transportation arrangements. This decentralized approach will bridge the gap in ANC accessibility, ensuring that even those in remote settings receive timely and adequate care (19).

#### 3.2.2 Telemedicine for ANC consultations

To overcome geographical barriers and enhance ANC quality, there is a need to pilot and scale up telemedicine initiatives. This involves leveraging technology to connect pregnant individuals in remote areas with skilled healthcare providers for virtual consultations. This innovative approach not only improves the reach of ANC services but also facilitates early identification of high-risk pregnancies, minimizing complications during labor and delivery (20). While telemedicine for ANC consultations holds promise, as evidenced by successful programs in countries like Zimbabwe, South Africa (21, 22), its applicability in Zambia requires careful consideration. Limited internet connectivity in remote areas presents a significant hurdle (23). However, pilot programs in areas with better infrastructure can be implemented to assess effectiveness and gather data to inform future expansion plans. Zambia's growing smartphone penetration rate indicates a potential user base for mobile health applications used in telemedicine (24). It's important to acknowledge that data privacy and security measures need to be established for these consultations. Additionally, training healthcare workers on effectively conducting telemedicine consultations would be crucial for successful implementation.

### 3.3 Enhancing intrapartum care

To reduce PPH during delivery, there should be correct and consistent use of the partogram, as well as constant availability of uterotonic drugs.

#### 3.3.1 Correct and consistent use of partogram

Ineffective monitoring during labor contributes to delays in identifying deviations from normal progression, leading to increased PPH cases. In a study conducted to determine the impact of maternal death reviews in the country, partogram use and/or interpretation errors were identified as challenges associated with maternal deaths (1). To address this, the Ministry of Health should emphasize continuous mentorship for healthcare staff, including midwives and students, in the correct and consistent use of the partogram. This tool aids in timely intervention, such as the augmentation of labor or cesarean sections, thereby preventing complications and reducing the incidence of PPH (25).

#### 3.3.2 Sustained availability of uterotonic drugs

Because prophylactic uterotonic drugs can prevent PPH, they are routinely advised. They cause the uterus to contract, which lessens excessive bleeding after birth. A few of the uterotonic drugs that are used are carbetocin, misoprostol, and oxytocin (26). A cross-sectional study conducted in Kenya, Uganda, and Zambia revealed that availability of either oxytocin or misoprostol at healthcare facilities for the management of PPH in Zambia in 2017 was 76% (27). Ensuring the availability of uterotonic drugs is crucial for the effective management of PPH (28). The Ministry of Health should strengthen the commodity security of uterotonics such as oxytocin, tranexamic acid, and misoprostol. This strategic focus aims to guarantee that these essential drugs are readily accessible across all medical facilities, regardless of their geographical location, ensuring prompt and effective intervention in cases of PPH (27).

### 3.4 Training and equipment for PPH management

Healthcare professionals should be trained in emergency obstetric care, the use of anti-shock garments, and balloon tamponade so that they can manage PPH appropriately.

#### 3.4.1 Use of anti-shock garments

Training healthcare workers in the use of the anti-shock garment is a vital aspect of PPH management. Although the availability of these garments poses a challenge due to their cost, a study conducted in Zambia and Zimbabwe revealed that the use of anti-shock garments was cost-effective in Zambia (29). The Ministry of Health should actively explore avenues to address the financial constraints associated with acquiring and maintaining these life-saving devices. Ensuring widespread availability and proper training can significantly enhance the capacity to manage PPH at various levels of care (30).

#### 3.4.2 Emergency obstetric care (EMoC) training

Empowering midwives and medical doctors with emergency obstetric care training is fundamental to managing obstetric emergencies effectively. The Ministry of Health should invest in scaling up specialized training for midwives and medical doctors, enabling them to handle a range of obstetric complications promptly. This initiative aligns with the broader goal of decentralizing emergency obstetric care, ensuring that timely interventions are possible at different healthcare facilities (31). Existing efforts to expand the healthcare workforce can be categorized into three main approaches. The first strategy focuses on increasing training capacity by partnering with universities and colleges to produce more healthcare professionals. Secondly, decentralizing training programs brings education closer to where healthcare workers are needed, improving accessibility. Most recently, e-learning platforms have emerged as a method to deliver training materials online, potentially reaching a wider audience at a lower cost. These combined efforts aim to achieve a larger pool of skilled providers, leading to improved patient outcomes and a reduction in healthcare access disparities. While the full impact of these approaches is still being evaluated, the initial results hold promise for a more robust healthcare workforce.

#### 3.4.3 Training for medical doctors in balloon tamponade

While medical doctors are essential for managing severe PPH cases, a critical skill gap exists in Zambia regarding the use of balloon tamponade, a lifesaving procedure (32, 33). Recognizing this, the Ministry should invest in scaling up training programs to equip medical doctors with the knowledge and skills required for the correct application and removal of balloon tamponade. This intervention is crucial for averting life-threatening complications associated with severe PPH (32).

#### 3.4.4 Clinical mentorship programs

To further enhance the skillset of healthcare professionals managing PPH, particularly at lower-level facilities, the Ministry of Health should increase its investment in targeted clinical mentorship programs. Obstetrics and gynaecology specicialists, midwives, and anesthesiologists can offer dedicated, on-site mentorship to doctors and nurses responsible for PPH management. These programs could focus on areas like managing blood loss, applying anti-shock garments, performing specific procedures like uterine tamponade, and interpreting clinical signs. Regular sessions (e.g., monthly) over a sustained period (e.g., 6 months) would facilitate knowledge transfer and skill development. Studies have shown that clinical mentorship programs can significantly improve maternal health outcomes, including reducing PPH-related mortality (34, 35). The Government of Ethiopia's national RMNCH mentorship program, which includes clinical mentorship for midwives and nurses, has shown promise in improving the quality of maternal and newborn care (35). Investing in such programs aligns with the overall strategy of decentralizing emergency obstetric care and ensuring timely access to quality care throughout the healthcare system. To ensure successful implementation, collaboration with professional organizations, securing funding for specialist travel, and addressing workload concerns are crucial aspects to consider.

## 4 Conclusion

The comprehensive approach undertaken by the Ministry of Health in Zambia reflects a commitment to addressing the complex challenges associated with PPH. While commendable progress has been made in blood transfusion services, surgical skills development, and antenatal care, gaps remain. Strengthening infrastructure and resource mobilization for blood services, expanding surgical training through task shifting and community engagement, intensifying community-based ANC efforts, and continuously enhancing intrapartum care – from labor monitoring to emergency obstetric training – are key opportunities for further improvement.
